# Do Interlimb Knee Joint Loading Asymmetries Persist throughout Stance during Uphill Walking Following Total Knee Arthroplasty?

**DOI:** 10.3390/ijerph20146341

**Published:** 2023-07-11

**Authors:** Tanner Thorsen, Chen Wen, Jared Porter, Jeffery A. Reinbolt, Joshua T. Weinhandl, Songning Zhang

**Affiliations:** 1 School of Kinesiology and Nutrition, The University of Southern Mississippi, Hattiesburg, MS 39406, USA; 2 Department of Kinesiology, Recreation and Sport Studies, The University of Tennessee, Knoxville, TN 37996, USA; 3 Department of Mechanical, Aerospace, and Biomedical Engineering, The University of Tennessee, Knoxville, TN 37996, USA; reinbolt@utk.edu

**Keywords:** total knee arthroplasty, musculoskeletal modeling, knee compressive force, uphill walking

## Abstract

The purpose of this study was to determine differences in total (TCF), medial compartment (MCF), and lateral compartment (LCF) tibiofemoral joint compressive forces and related muscle forces between replaced and non-replaced limbs during level and uphill walking at an incline of 10°. A musculoskeletal modeling and simulation approach using static optimization was used to determine the muscle forces and TCF, MCF, and LCF for 25 patients with primary TKA. A statistical parametric mapping repeated-measures ANOVA was conducted on knee compressive forces and muscle forces using statistical parametric mapping. Greater TCF, MCF, and LCF values were observed throughout the loading response, mid-stance, and late stance during uphill walking. During level walking, knee extensor muscle forces were greater throughout the first 50% of the stance during level walking, yet greater during uphill walking during the last 50% of the stance. Conversely, knee flexor muscle forces were greater through the loading response and push-off phases of the stance. No between-limb differences were observed for compressive or muscle forces, suggesting that uphill walking may promote a more balanced loading of replaced and non-replaced limbs. Additionally, patients with TKA appear to rely on the hamstrings muscle group during the late stance for knee joint control, thus supporting uphill walking as an effective exercise modality to improve posterior chain muscle strength.

## 1. Introduction

It is projected that over the next several decades, the incidence of total knee arthroplasty (TKA) will grow over 400% [1]. The primary goals of TKA are to alleviate knee pain and restore the loss of knee joint functions [2,3]. Patients with TKA have reported great difficulty during daily tasks such as getting out of bed, ascending stairs, shopping, and walking [4,5]. 

Although it has been incorporated in exercise and rehabilitative routines, one daily task those with TKA may encounter is uphill walking [6]. This may come in the form of ramp negotiation or recreational hiking activities [7]. Wen et al. (2019) conducted one of the first biomechanical studies of uphill walking in which patients with TKA and heathy controls performed walking trials on slopes of 0° (level), 5°, 10°, and 15° [8]. Patients with TKA reported greater knee pain during all walking conditions compared to the healthy control participants. They also exhibited a lower internal knee extension moment in both the replaced and non-replaced limbs than did healthy controls. More importantly, there was a significant interaction between the limb and slope, showing that the non-replaced limb demonstrated greater increases in peak knee extension moment from 0° to 15° than the replaced limb. However, Wen et al. did not investigate tibiofemoral compressive forces, and more comprehensive investigations of tibiofemoral joint loading during uphill walking in people with TKA may help to inform rehabilitation protocols and prosthesis design [8].

Obtaining true tibiofemoral compressive forces in vivo requires the use of specialized instrumented prostheses, which can be very costly and not practical for large-scale use. Furthermore, these instrumented prostheses only report forces in the replaced limb, and not in the contralateral, non-replaced limb, making between-limb comparisons impossible. Utilizing musculoskeletal modeling and simulation techniques, we recently demonstrated that peak tibiofemoral compressive forces between limbs (replaced vs. non-replaced) of patients with TKA may be more symmetrical between the replaced and non-replaced limbs than other joint kinetic variables have typically reported, and that different strategies in movement patterns may allow for this more balanced loading to occur [9]. Though these discrete instances of peak loading at the knee were not statistically significant between limbs during uphill walking, the question of total stance-phase joint loading patterns was still of particular interest in developing a better understanding of how the knee joint mitigates loads during uphill walking activities for TKA patients. This has implications for rehabilitation and post-TKA activities that are prescribed or recommended to patients. 

With an eye toward postoperative care and the return to more “normal” life in mind, we aimed to examine differences in compartment-specific tibiofemoral compressive forces (total compressive force (TCF), medial compartment compressive force (MCF), and lateral compartment compressive force (LCF)) and related knee-joint-spanning muscle forces between different limbs (replaced, non-replaced) and different slopes (0° (level), and 10° (uphill)) throughout the stance phase. Based on our previous work [9], we hypothesized that stance-phase compressive forces would be similar between the replaced and non-replaced limbs, and that we would expect to see a significant difference in compressive forces due to the slope of uphill walking. 

## 2. Materials and Methods

### 2.1. Participants

Twenty-five patients with TKA (27.8 ± 3.2 months since surgery, male: 11, female: 14, age: 68.6 ± 4.9 years, height: 170.0 ± 11.0 cm, mass: 83.2 ± 15.6 kg) were recruited from a local orthopedic clinic to attend one laboratory session. Of our sample of twenty-five patients, all had received a primary TKA from the same orthopedic surgeon within 5 years of participation. Participants received either cruciate retaining (*n* = 10, Journey II, Smith & Nephew, Memphis, TN, USA), bi-cruciate retaining (*n* = 5, Journey II, Smith & Nephew, Memphis, TN, USA), or posterior stabilized implants (*n* = 10, Persona, Zimmer, Warsaw, IN, USA). Inclusion/exclusion criteria and full data collection methods have been previously reported [8]. In short, inclusion criteria were having a primary unilateral TKA from the same surgeon between 6 and 60 months prior to participating in this study. Patients were excluded if they had received any other lower-extremity joint arthroplasty; any diagnosed osteoarthritis of the hip or ankle; more than 75% radiographic joint space narrowing and chronic pain of the contralateral, non-replaced knee; BMI greater than 38 kg/m^2^; or any neurological diseases. Prior to participation in the study, all participants provided informed consent approved by the University’s Institutional Review Board.

### 2.2. Experimental Protocol

All participants completed five trials of uphill walking at a self-selected pace on 0° (level walking) and 10° inclines on a customized adjustable ramp system that was instrumented with two force platforms. A trial was deemed successful if contact was made with only the force plate during the ramp ascent or level walking. To minimize the duration of the data collection session, ramp incline conditions were performed first, followed by the level walking conditions. 

### 2.3. Instrumentation

Three-dimensional (3D) kinematics (240 Hz, Vicon Motional Analysis Inc., Oxford, UK) and the ground reaction force (GRF, 1200 Hz, BP600600 and OR-6-7, American Mechanical Technology Inc., Watertown, MA, USA) were simultaneously recorded. To define each anatomic segment, passive reflective markers were placed bilaterally on each participant’s acromion process, iliac crest, greater trochanter, medial and lateral femoral epicondyles, medial and lateral malleoli, the distal phalanx of the second toe, as well as the heads of the first and fifth metatarsals. Rigid-body marker clusters were used to track the motion of each segment during walking trials and were placed on the trunk, pelvis, thighs, shanks, and feet.

A 16-channel surface electromyography (EMG) system (1200 Hz, Trigno™ Wireless EMG System, Delsys, INC, Natick, MA, USA) was used to record muscle EMG activations bilaterally on the following muscles: vastus lateralis, vastus medialis, medial head of the gastrocnemius, semitendinosus, and biceps femoris. The site of electrode attachment was cleaned and shaven prior to the application of electrodes. The placement of the EMG electrodes on the selected muscles was based on the recommendations of SENIAM [10]. Kinematic, GRF, and EMG data were sampled simultaneously (2.5, Vicon Motion Analysis Inc., Oxford, UK).

### 2.4. Musculoskeletal Modeling and Simulation

An open-source musculoskeletal model (18 segments, 23 degrees of freedom (DOFs), 92 muscle–tendon actuators) was used to perform the simulations [11]. The knee joint of this model consists of 1 degree of freedom (sagittal plane rotation) partitioned to create both medial and lateral compartments. The model was scaled for each participant, and subtalar and metatarsal–phalangeal joint rotations were locked. 

Generalized joint coordinates derived from inverse kinematics calculations were exported from Visual3D (Version 6, C-Motion, Inc., Germantown, MD, USA) and imported into OpenSim for simulations (3.3 OpenSim, SimTK, Stanford University, Stanford, CA, USA). The generalized joint coordinates were applied to each subject-specific scaled musculoskeletal model. Inverse dynamics calculations were performed in OpenSim to compute lower-extremity joint moments. Next, muscle activations and forces were calculated using static optimization [12,13]. The static optimization calculations included muscle physiology (force–length–velocity relationships) and an objective function to minimize the sum of squared muscle activations [13]. Maximum reserve torque actuator values for all lower-extremity joints were checked and found to be within suggested guidelines [14]. Joint compressive forces (MCF, LCF, TCF) were calculated using the joint reaction analysis tool in OpenSim and expressed in the tibia reference frame [15]. 

Raw EMG signals were band-pass filtered at cutoff frequencies of 10 Hz and 450 Hz and then full-wave rectified. A moving root-mean-square (RMS) filter was used to filter the rectified EMG signals using a 60-millisecond moving window. The maximum value of the RMS EMG signals of three functional test trials was used to normalize the filtered EMG signals of the testing movement trials. 

Primary variables of interest included TCF, MCF, and LCF. Muscle forces of the knee extensors and flexors were also included as secondary variables. The knee extensors group includes the rectus femoris, vastus lateralis, vastus intermedius, and vastus medialis. The flexors include the biceps femoris long and short heads, semimembranosus, semitendinosus, sartorius, gracilis, and both medial and lateral heads of the gastrocnemius. All variables of interest were analyzed during the stance phase of the gait cycle, defined as the interval between the vertical GRF crossing a threshold of 10 N (heel-strike) and subsequently falling below the threshold (toe-off), as measured by in-ground force platforms. 

### 2.5. Statistical Analysis

To examine differences in tibiofemoral compressive and muscle forces between limbs and slopes, one-dimensional statistical parametric mapping (SPM) using Random Field Theory to correct for Type I error inflation [16,17] was implemented using MATLAB (R2019B, MathWorks, Natick, MA, USA) with the source code made available by Pataky et al. [17]. 

Compressive and muscle forces were compared using a 2 × 2 (limb (replaced, non-replaced) × slope (level, 10°)) SPM repeated-measures ANOVA. Limb and slope main effects were deemed significant when the SPM trajectory crossed the critical threshold [16]. If a significant limb × slope interaction was found, post hoc SPM{t} tests were conducted on each pairwise comparison. The magnitude of effect for all significant post hoc comparisons was determined by computing the mean difference between the two signals throughout the duration of time the SPM trajectory was above the critical threshold and was reported using Cohen’s d [18,19,20]. 

## 3. Results

**Figure 1:**
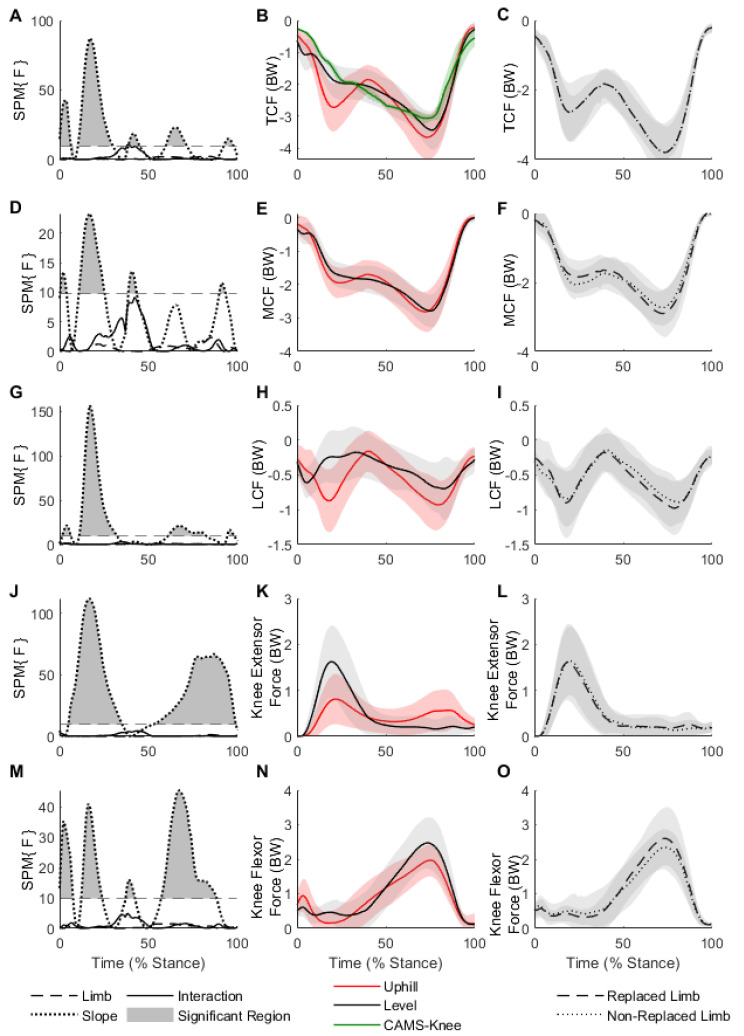
(**A**–**C**) SPM results, waveforms for TCF during uphill and level walking, and waveforms for replaced and non-replaced limbs during uphill walking, respectively, (**D**–**F**) SPM results, waveforms for MCF during uphill and level walking, and waveforms for replaced and non-replaced limbs during uphill walking, respectively, (**G**–**I**) SPM results, waveforms for LCF during uphill and level walking, and waveforms for replaced and non-replaced limbs during uphill walking, respectively, (**J**–**L**) SPM results, variable waveforms for Knee Extensor muscle force during uphill and level walking, and waveforms for replaced and non-replaced limbs during uphill walking, respectively, and (**M**–**O**) SPM results, variable waveforms for Knee Flexor muscle force during uphill and level walking, and waveforms for replaced and non-replaced limbs during uphill walking, respectively. Regions of statistical significance for the SPM test are indicated by dark shaded areas. The waveforms presented in columns 2 and 3 indicate the mean ± 1 standard deviation (red or gray shaded region).

The TCF demonstrated a significant main effect of slope, indicating different TCFs between uphill and level walking during five separate stance intervals (Figure 1A,B, Table 1). During the first 30% of the stance phase, the TCF was greater during uphill walking with a mean difference of 0.68 BW (Figure 1A,B, Table 1). During the push-off phase, the TCF was still greater during uphill walking, only by a mean difference of 0.34 BW, however.

During the first 26% of the stance phase, the MCF was greater during uphill walking with a mean difference of 0.25 BW (Figure 1D,E, Table 1). During push-off, the MCF was still greater during uphill walking, only by a mean difference of 0.14 BW, however. The SPM test for LCF also revealed a significant main effect of slope (Figure 1G,H, Table 1). During the first 33% of the stance phase, the LCF was greater during uphill walking with a mean difference of 0.41 BW, and preparing for push-off when walking uphill, the LCF was greater by a mean difference of 0.23 BW. 

Both SPM tests for the knee-joint-spanning muscle forces revealed a significant main effect of slope. The knee extensor muscle forces were significantly lower during uphill walking during the loading response, by an average mean difference of 0.53 BW, yet greater by 0.24 BW during push-off when walking uphill (Figure 1J,K, Table 1). Knee flexor muscle forces demonstrated reduced muscle force in both loading response and push-off while walking uphill by 0.27 and 0.42 BW, respectively (Figure 1M,N, Table 1).

**Figure 2:**
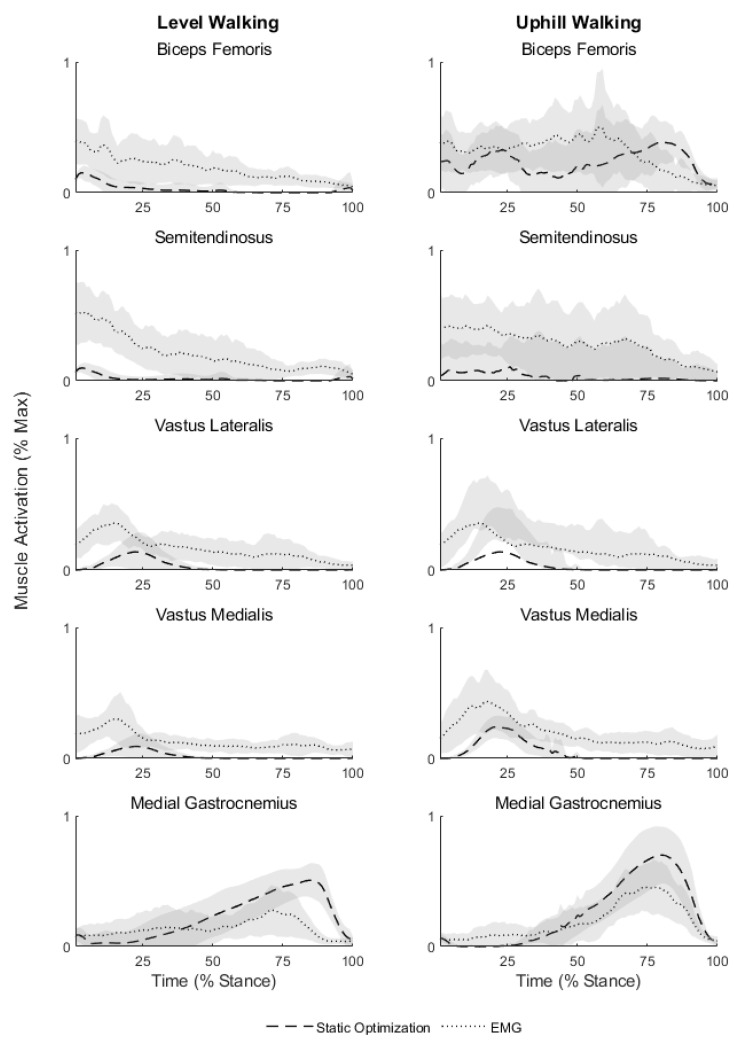
Muscle activation patterns of the replaced limb during level and uphill walking. Shaded regions represent ±1 standard deviation.

The frontal-plane lower limb alignment was determined by calculating the mechanical axis angle using motion capture data [21,22,23]. The mechanical axis angle was found to be similar between limbs. Reserve torque actuators for all lower-extremity joints were manually inspected and found to be within suggested guidelines [15]. There was fair agreement in the trends of the experimentally collected EMG and the model-predicted muscle activations (Figure 2). Although activation patterns were consistent for most muscles included in our analysis, the biceps femoris muscle during uphill walking and the medial gastrocnemius muscle during the late stance in both level and uphill walking seemed to be favored by the model compared to experimentally recorded EMG signals.

## 4. Discussion

We sought to investigate differences in tibiofemoral joint compressive forces (TCF, MCF, LCF) and knee-joint-spanning muscle forces between different limbs (replaced, non-replaced) and different slopes (0° and 10°) through the entirety of the stance phase. Our hypothesis was supported, in that no between-limb differences were observed at either slope and that the slope significantly impacted both compressive and muscle forces.

Results for the TCF showed no differences between the replaced and non-replaced limbs. Inverse-dynamics-based studies have frequently used the internal knee extension to indicate overall loading at the knee joint [24,25,26,27,28,29]. For the TKA population specifically, overall joint loading is of particular interest as it has been related to increased wear and degradation of the prosthesis and joint loading asymmetry [25,26]. Previous studies have demonstrated a deficit in knee extension moment in the replaced limb compared to the non-replaced limbs of patients with TKA in various activities such as level walking, stair ascent, and ramp ascent [8,30,31]. The between-limb symmetry of the TCF found in this current study may be influenced by forces produced by knee-joint-spanning muscles. The magnitude of the TCF is contributed from three sources: GRF, muscle forces, and the inertial characteristics of the segment [15]. During level walking, the peak vertical GRF has been reported to be similar with 1.08 body weights (BW) for healthy limbs of controls, and 1.04 BW for the non-replaced limb and 1.03 BW for the replaced limbs of patients with TKA [8]. While inertial characteristics of the limb contribute minimally to the compressive forces, muscle forces are the primary contributor to the TCF. In this current study, knee extensor muscle forces range between 1.5 and 2.0 BW, and knee flexor muscle forces range between 2.0 and 3.0 BW (Figure 1). Although not statistically significant between limbs, the knee flexor muscle forces produced over 2.0 BW of force during push-off. Given the lack of between-limb significance in this study, it is possible that different gait strategies have been adopted by individual patients that occlude significant between-limb differences in this sample. Some patients with better post-operative recovery may exert greater or equal amounts of knee extensor and flexor muscle forces in the replaced limb during walking, while others may rely more heavily on muscle forces from the non-replaced limb.

Similar trends in statistical significance throughout the stance phase were observed for the MCF and LCF, indicating a shift in compressive forces from the medial compartment to the lateral compartment when walking uphill. The change in magnitude of compressive force between level and uphill walking was substantially higher in the LCF than in the MCF. Both loading response and push-off TCF were larger in uphill walking, and the magnitude of the TCF seems to be in part influenced by the shift in compressive-force-off of the medial compartment and onto the lateral compartment of the knee. Given the nature of increased medial compartment joint loading (i.e., increased MCF) that was likely a contributing factor to knee osteoarthritis (OA) preceding TKA, these findings may be encouraging for uphill walking as a means to redistribute joint loads off of the medial compartment, and onto the lateral compartment. Though not statistically significant, a small decrease in loading response MCF was observed in the replaced limb (Figure 1F). Qualitatively, this aligns with the literature that has shown a decreased peak MCF in the replaced limb following TKA. Using regression equations first determined by Walter et al. [32], Wen et al. [8] estimated the peak MCF of replaced and non-replaced limbs by using a combination of knee extension moment and peak knee abduction moment and reported a greater loading response MCF in the non-replaced limb at an incline of 10°.

Significant differences in knee extensor muscle force were present during both the loading response and push-off between slopes (Figure 1J). An increased loading-response knee extensor muscle force with changing incline is a logical expectation that would be in-line with the significant changes seen in the TCF. However, during the loading response, the knee extensor muscle force was greater during level walking (Figure 1K), yet the TCF was greater during uphill walking (Figure 1B). Knee extensor muscle force is a primary contributor to the TCF—in addition to the GRF and segment inertial properties. Li et al. [33] also reported reduced quadriceps muscle forces during the loading response, which culminated in a reduced knee extension moment during level walking. With the increased demand of the uphill walking task, an avoidance of the quadriceps muscles likely suggests that participants are utilizing alternative gait strategies to control knee flexion and joint loading, thus reducing muscular force demands surrounding the knee joint during both level and uphill walking tasks [33].

A secondary finding of this study is that changes to tibiofemoral joint compressive forces between slopes occur specifically during the loading response. Uphill walking increased the TCF, MCF, and LCF on average by 0.68, 0.25, and 0.41 BW, respectively, during the loading response phase. The knee extensors produce eccentric force to absorb loading to the knee joint and maintain posture, but quickly transition to concentric force production to extend the knee joint. In uphill walking, the TCF increased with slope during the loading response (specifically 12–30% of stance); however, the knee extensor muscle force was lower than during level walking. Given the increase in loading response TCF during uphill walking that is not accompanied with a significant increase in knee extensor muscle forces, it is likely that dynamic control of the knee joint may be coupled through other joints such as the hip or ankle. Though knee-joint-spanning muscles cannot solely explain the behavior of the TCF, uphill walking may promote an environment that especially engages the quadriceps muscles during push-off without consequential increases in joint loads compared to level walking (Figure 1K), offering further support of uphill walking in rehabilitative and re-strengthening protocols.

Wen et al. recommended against the use of 10° and 15° uphill walking during TKA rehabilitation due to an increased internal knee extension moment experienced by the replaced limb, and the association between knee extension moment, increased TCF, and damage to the knee prosthesis [8,34,35]. Deficits in quadriceps strength and knee extension moment in the replaced limb have been demonstrated immediately following TKA operation up to several years post TKA [36,37]. Recent recommendations, however, have suggested that, despite deficits in the replaced limb knee extension moment, early high-intensity rehabilitation following TKA leads to improved short-term and long-term functional outcomes compared to a lower-intensity rehabilitation program [38,39,40]. As a part of both high- and low-intensity rehabilitation programs, quadriceps strengthening exercises such as quadriceps setting, weight bearing lunges, body-weight squatting, and stair ambulation have been incorporated into rehabilitation plans for patients with TKA to improve muscle strength asymmetries between the replaced and non-replaced limbs [39]. However, quadriceps strengthening has been shown to have no effect on the internal knee extension or abduction moments in patients with knee osteoarthritis in gait [41,42,43]. In this context, uphill walking may be an effective exercise for high-intensity early and long-term rehabilitation programs, with a lower peak GRF than stair ambulation. Additionally, uphill walking facilitates increased muscular demand and quadriceps strengthening with increased slope while promoting the reacquisition of normal gait patterns following TKA, which may not be achieved in traditional quadriceps strengthening exercises.

There are certain limitations to this work that need to be acknowledged. SPM analysis between groups or conditions mandates temporal synchrony for comparisons over time to be made. In order to meet these requirements, time-normalization (to 101 data points) was performed on compressive and muscle force waveforms. With such reductions in resolution, it is possible that true peak values may be reduced (or smoothed out) as a result of the time normalization, which may mute observable differences of compressive or muscle forces between limbs. However, we feel that the consequences of this time normalization were minimal given our objective of comparing between conditions within our sample.

Lerner et al. reported contact force estimations using three variations of this model [11]. The fully informed model, using both frontal plane lower-limb alignment and condylar contact points, produced the best estimation of compressive force. In these current data, condylar contact locations were unknown. Thus, we utilized motion capture data of the static standing trial to estimate lower limb alignment [22,23]. With comparable frontal plane alignment (Table 1), we feel confident that any differences that may arise from implementing participant-specific frontal plane lower-limb alignments were minimized.

## 5. Conclusions

Joint loading appears to be similar between replaced and nonreplaced limbs during uphill and downhill walking. Due to the increased difficulty of the uphill walking task, the TCF and MCF were shown to increase with slope, while the loading response knee extensor muscle force and push-off knee flexor muscle forces were greater during level walking, suggesting the adoption of alternative gait strategies to reduce muscular demand at the knee following TKA. Uphill walking may be an effective exercise for high-intensity early and long-term rehabilitation programs, promoting increased muscular demand and quadriceps strengthening while encouraging the reacquisition of normal gait patterns following TKA.

## Figures and Tables

**Table 1:** Statistically significant regions as identified by SPM between level and uphill walking for all knee joint compressive and muscle forces. Significant regions (% stance), *p*-values of each associated significant region, and mean difference between level and uphill walking within each significant region (BW).

	Region	*p*	Mean Difference
TCF	1–9%	0.016	0.25
	12–30%	<0.001	0.68
	40–46%	0.017	0.21
	61–73%	0.001	0.34
	95–99%	0.024	0.13
MCF	1–5%	0.038	0.18
	12–26%	<0.001	0.25
	40–45%	0.031	0.14
	91–95%	0.039	0.14
LCF	1–7%	0.032	0.13
	12–33%	<0.001	0.41
	62–84%	<0.001	0.23
	96–99%	0.0038	0.08
Knee Extensor Muscle force	6–36%	<0.001	0.53
	54–100%	<0.001	0.24
Knee Flexor Muscle force	1–8%	0.018	0.27
	14–25%	0.004	0.27
	38–44%	0.031	0.19
	58–89%	<0.001	0.42

## Data Availability

The data presented in this study are available on request from the corresponding author. The data are not publicly available.
